# Factors associated with high use of general practitioner and psychiatrist services among patients attending an addiction rehabilitation center

**DOI:** 10.1186/s12888-016-0974-7

**Published:** 2016-07-22

**Authors:** Christophe Huỳnh, André Ngamini Ngui, Sylvia Kairouz, Alain Lesage, Marie-Josée Fleury

**Affiliations:** Centre de recherche et d’expertise en dépendance du Centre intégré universitaire de santé et des services sociaux du Centre-Sud-de-l’Île-de-Montréal, 950 Louvain East, Montréal, Québec H2M 2E8 Canada; Douglas Mental Health University Institute, McGill University, 6875 LaSalle Boulevard, Montréal, Québec H4H 1R3 Canada; Department of Sociology and Anthropology, Concordia University, 1455 de Maisonneuve Boulevard West, Montréal, Québec H2G 1M8 Canada; Centre de recherche Fernand-Seguin, Institut universitaire en santé mentale de Montréal, 7401 Hochelaga Street, Montréal, Québec H1N 3M5 Canada

**Keywords:** Health service use, Substance use disorders, High users of health services, Substance abuse treatment center, Addiction rehabilitation center, General practitioners, Psychiatrists

## Abstract

**Background:**

This study aimed to 1) identify the characteristics of individuals with substance use disorders (SUDs) who make high use of services provided by general practitioners (GP) and psychiatrists while receiving services concurrently from an addiction rehabilitation center (ARC), and 2) to compare high service users to moderate and low service users.

**Methods:**

Data were compiled for 4,407 individuals with SUDs who were receiving services from an ARC in 2004. The data came from the merging of four databases: the ARC data registry (January 1^st^, 2004–December 31, 2004), the Quebec Health Insurance Board database (March 31, 2003–April 1st, 2005), the Quebec provincial database for hospitalizations (March 31, 2003–April 1st, 2005), and the Quebec National Institute of Public Health database (2004). Independent variables were grouped according to the Andersen Behavioral Model of Health Services Use: predisposing, enabling and need factors. Generalized estimating equations analyses were performed to assess the influence of individual and neighborhood-level characteristics on high use of services outside the ARC provided by GPs and psychiatrists. Benjamini-Hochberg’s procedure was applied to correct for multiple comparisons.

**Results:**

About 97 % of individuals attending the ARC consulted a GP or a psychiatrist during the two-year study period, for a mean of 1.5 consultations per month. Findings revealed that 5 % of the sample made 26 % of all consultations over the two years, and they were defined as high users. No single predisposing factor was associated with high use. One enabling factor significantly increased the risk of being a high user of services from general practitioners and psychiatrists: receiving services at the ARC for three years prior to 2004. Four needs factors, all related to mental health diagnoses (schizophrenia, mood disorder, anxiety disorder, personality disorder), predicted high use of general practitioner and psychiatrist services.

**Conclusions:**

This study found that nearly all individuals with SUDs receiving services from an ARC were users of health services from GPs and psychiatrists outside the ARC. High users most probably accessed them in inpatient settings. No previous study has compared high service users with low and moderate users among individuals with SUDs. Considering that ARCs are treating individuals with complex needs, some of whom make high use of medical professionals, both ARCs and their clients could benefit from increased collaboration and integration between the addictions and mental healthcare sectors.

## Background

Substance use disorders (SUDs), including alcohol and drug abuse and dependence, are associated with adverse medical and social consequences such as cardiovascular diseases, cancers, suicidal behaviors, injuries, and crime [1, 2]. They often co-occur with other mental disorders, such as schizophrenia, mood disorders, anxiety disorders and personality disorders [3–6]. The recurrent nature of SUDs and their association with co-occurring conditions may incite individuals with SUDs to use a plethora of services provided by medical professionals, in addition to attending addiction rehabilitation centers (ARCs). In a context of resource constraint, it is important to examine the determinants of high service use among these individuals.

Although North American and European epidemiological studies report that about 10 % of individuals with SUDs use health services [7–10], clinical research shows that they represent over 25 % of the cases seen in the health system [11, 12]. Studies on individuals with SUDs treated in ARCs report that 27–61 % have used a hospital emergency department; 12–56 % have been hospitalized; 20–85 % have used some type of outpatient setting (for example, a physician outpatient clinic); and 13–65 % have received some form of mental health services [13–27]. Health service use by individuals with SUDs outside of ARCs can be explained by the fact that most medical professionals, such as general practitioners (GPs) and psychiatrists, are working in clinical settings such as hospitals and outpatient clinics/offices [21, 27]. SUDs require long-term care that is generally provided by GPs in primary care settings who refer clients to specialized care, as needed [28–31]. Given the high prevalence of co-occurring SUD and other mental disorders [3–5, 15, 19], psychiatrists are also important service providers for this population [32–34]. One study conducted with 615 heroin users in an Australian ARC indicated that 60 % of individuals with SUDs had consulted a GP and 7 % consulted a psychiatrist in the preceding month [35].

Individuals with SUDs require different types of services with varying intensity to meet their needs. As such, some individuals, called high service users, are frequent and intense users of a variety of services. Service needs among high users of healthcare services present challenges in the present context of increasing resource constraint. While no consensus exists in the literature on the definition of high service use, three types of threshold are often used in scientific research: proportions (percentile, percentage), absolute numbers (number of consultations), and parametric values (standard deviation) [36].

Several studies have identified socio-demographic and clinical characteristics associated with high service use among individuals with SUDs who are treated in ARCs [37, 38]. However, these factors have mainly concerned health services use within ARCs and emergency departments as opposed to the services provided by medical professionals. Injection drug users who are frequent users of ARC, defined as those falling within the 90–95^th^ percentile of consultations (i.e. the top 5–10 % of consultations), are more likely than less frequent users to receive other types of treatment. They also tend to be older, male, to have been homeless at some point in their lives, to have a history of mental health treatment, to have a criminal record, to have injected drugs in the past month, and used other programs in addition to detoxification [39, 40]. A study in California identified several variables that were associated with high use of mental health and substance abuse services, defined as three or more consultations in a given fiscal year, namely age (over 45 years old), sex (female), ethnicity (Caucasian or Afro-American), homelessness, bipolar disorder, schizophrenia or other psychotic disorders, and having insurance [41]. Among individuals in Quebec with co-occurring SUDs and schizophrenia, high users of emergency departments, defined as those falling within the 95^th^ percentile of annual consultations, were found to be younger, more likely to live in rural areas or in regions peripheral to a university-affiliated hospital, more frequently hospitalized, and to have a greater number of comorbid physical and psychiatric conditions as compared with moderate or low users [42]. Taken together, these studies show that high users of health services are characterized by greater needs and more complex clinical conditions regardless of how “high use” is defined.

This is the first study to identify the characteristics of high users of services provided by GPs and psychiatrists among individuals with SUDs attending an ARC. High service users are compared with moderate and low users according to Andersen’s behavioral model, a conceptual framework that has been extensively applied in research on health service utilization [43, 44]. Understanding the factors associated with high use of services provided by GPs and psychiatrists among individuals with SUDs may help improve healthcare organization, and increase our ability to respond adequately to the needs of this population. It is expected that higher needs related to mental conditions will determine higher use of GPs and psychiatrists among individuals with SUDs who receive treatment at an ARC.

## Methods

### Study setting

This study was conducted in the largest ARC in the province of Quebec (Canada). Located in Montreal, the facility is specialized in evaluation, treatment and rehabilitation of francophone individuals dealing with SUDs and compulsive gambling. Individuals with gambling addiction, which often co-occurs with SUDs, represent a relatively small proportion of service users [45, 46]. Programs include group activities, services for homeless people, support for social integration, residential rehabilitation, detoxification programs, and crisis services. Patients access these programs by self-referral or by referral from primary care professionals, including physicians, or the court. ARC services, as well as physician delivered care, are covered under Canada’s public health care system. The catchment area for this ARC includes the greater Montreal area, the second largest city in Canada with 1,988,243 inhabitants, and French-speakers represent approximately two thirds of the Montreal population [47].

### Procedure

The study used four large administrative databanks: 1) the ARC data registry (January 1^st^ to December 31, 2004), for client information on age, sex, place of residence, number of programs used in the ARC, and duration of attendance at the center; 2) physicians’ billing database of the *Régie de l’assurance maladie du Québec* (RAMQ) (March 31, 2003 to April 1st, 2005) for the number of consultations to medical professionals (GPs, psychiatrists, other specialists) as well as physician diagnoses for physical and mental conditions; 3) hospital discharge database, *Maintenance et exploitation des données pour l'étude de la clientèle hospitalière* (Med-Echo) (March 31, 2003 to April 1st, 2005), for information on hospital services provided (number and duration) and diagnoses for physical conditions and mental disorders given during hospital stay [48]; and 4) the *Institut national de la santé publique du Québec* database, for socio-geographic data on local community service centers (LCSC) based on territorial divisions in 2004. The LCSC is the smallest territorial level at which Quebec health and social services are organized [48].

The first step in data linkage was to obtain data from the two RAMQ and Med-Echo databanks for all individuals who received treatment at the ARC during the year 2004. Quebec citizens are provided with an individual identifier - the medical insurance number - within the health system. This number, also available in the ARC registry, was used to access user information in the RAMQ and Med-Echo systems. The list of ARC users by identification number was sent to the RAMQ and Med-Echo services to obtain data on their health care use. The RAMQ and the ARC databanks also included information on the LCSC territory in which users were residing in 2004. Socio-geographic data were obtained for each LCSC territory from the database of the *Institut national de la santé publique du Québec* [49]. Using SAS (Statistical Analysis System) software, a single user-level database was created by merging the information obtained from the four databases, to produce a single record for each service user. The merging of data from various sources was authorized by the Commission for Access to Information, and the study was approved by the research ethics board of the ARC.

### Variables

The dependent variable was the overall number of consultations to GPs and psychiatrists in any setting and for any reason, over the two-year period from March 31, 2003 to April 1^st^, 2005. Visits to GPs and psychiatrists in emergency departments and hospital inpatient units were also included in the count. Previous studies using data from the National Comorbidity Survey Replication have also included all contacts in any setting in calculating numbers of client consultations to GPs and psychiatrists [50, 51]. Moreover, a considerable number of health professionals in Canada work in these services: in 2004, it was estimated that 50.5 % of GPs also worked in hospital units and 23.5 % in emergency departments, in addition to their clinic [52].

In line with previous research on individuals with SUDs, high service users were defined as patients who fell within the 95^th^ percentile in terms of their number of consultations to GPs and psychiatrists (in other words, in the top 5 % of number of consultations) [39, 42]. On this basis, three categories of users were defined: high service users (95th percentile and over), moderate service users (26th to 94th percentile), and low service users (25th percentile or less). One of the advantages of this method is its use of standardized cut-off points for service utilization [42, 53]. The use of percentiles has been recommended instead of absolute values, as percentiles allow for more meaningful comparisons among various practices, countries and time periods [54].

Independent variables were grouped according to Andersen’s behavioral model [43, 44]. They included three categories of factors that may possibly confound or modify the determinants of health services use: these are predisposing, enabling and need factors [55–57]. According to the Andersen model, predisposing factors are individual characteristics prior to the onset of the illness. Enabling factors relate to structural factors and to the health system. Need factors include type of diagnosis and co-occurring disorders. In this study, all variables available from administrative databanks that fit with the Andersen model were used, allowing for a more comprehensive assessment of service use.

Predisposing factors included socio-demographic variables: sex and age. Enabling factors were characteristics defining the LCSC territory of residence: population density per square kilometer, proportions of individuals in the first (most affluent) and fifth (most deprived) quintile of material and social deprivation, social fragmentation (i.e. the sum of Z scores for number of privately rented households, number of single-person households, number of unmarried persons and number of persons who moved from one LCSC territory to another during the previous year) [58], percentage of men, proportion of each age group (up to 30 years old, 30–49 years old, 50–64 years old, 65 years old and above), proportion of recent immigrants in the past five years, distance from residence to ARC, and number of alcohol outlets in the territory. Enabling factors also encompassed variables on health service use, including frequency of consultations with medical specialists other than a psychiatrist, number of programs used in the ARC, duration of care episodes in the ARC, and duration of participation to ARC programs. Need variables consisted of diagnosis. All diagnoses were based on the World Health Organization’s International Classification of Diseases – 9^th^ or 10^th^ revision (ICD-9; ICD-10), and were given by the physician who assessed and/or treated the patient. SUDs (ICD-9: 291–305 and ICD-10: F10–F19), mental disorders, which include schizophrenia spectrum non-affective psychoses (ICD-9: 295; 297.1, 297.3; 298.8, 298.9; ICD-10: F20, F22-F24, F28, F29, F531) mood disorders (ICD-9: 296; 300.4; 311; ICD-10: F30-F34, F38, F39, F530), anxiety disorders (ICD-9: 300.0, 300.1, 300.2, 300.3, 300.5, 300.6, 300.7, 300.8, 300.9; 308.3; 309.0, 309.3, 309.4, 309.8, 309.9; ICD-10: F40-F43, F488, F489, F938), and personality disorders (ICD-9: 301; ICD-10: F60-F62, F68, F69, F21), as well as physical comorbidities (for complete list of ICD codes, see [59]). For the assessment of physical comorbidities, we used the Elixhauser comorbidity index [59], a validated method of the classification of comorbidities that is used primarily in the prediction of short- and long-term mortality.

### Statistical analyses

All analyses were performed using SAS 9.4. Depending on the type of variables, chi-square tests and t-tests were performed to compare high service users to low and moderate users. In order to control for intra-class correlations at the neighborhood level, we have used the generalized estimating equations (GEE) method rather than an ordinary least squares logistic regression to obtain robust standard errors estimators [60]. A multilevel approach was not possible because the sample size for some LCSC territories was too small to conduct this type of analysis. Thus, GEE were performed using the GENMOD procedure to assess factors associated with high use of GP and psychiatrist services, and to account for the nested nature of the data (i.e. individuals with LCSC territories). The Benjamini-Hochberg procedure was applied to correct for multiple comparisons. This procedure controls for the false discovery rate, which is the expected proportion of type I errors among the set of rejected null hypotheses [61, 62].

## Results

**Fig. 1:**
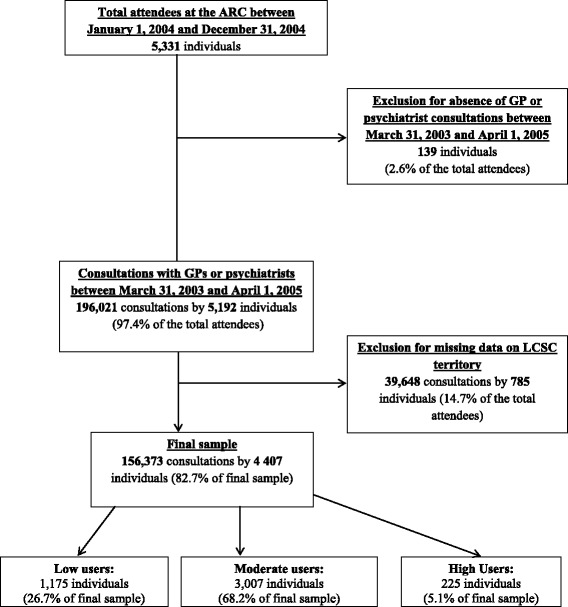
Flow chart diagram for the cohort selection (March 31, 2003 to April 1st, 2005)

As presented in Fig. 1, 139 (2.6 %) of the 5,331 individuals who consulted at the ARC in 2004 were excluded because they had not consulted any GP or psychiatrist. This exclusion procedure replicates that used in similar studies where only participants who had used a health service at least once were included [39, 42]. Another 785 ARC users with SUD (14.7 %) were excluded, because of missing information on their LCSC territory which does not allow for adequate testing using the Andersen model.

The final sample included 4,407 individuals (82.7 % of the initial sample) who consulted a GP or a psychiatrist over a two-year period. It was estimated that, for any medical reason, 56.4 % of our sample (*n* = 2,484) consulted GPs only, 43.2 % (*n* = 1,906) consulted both GPs and psychiatrists, and 0.4 % (*n* = 17) consulted psychiatrists only. It was not possible to carry separate analysis for each type of health professionals because of the low proportion of individuals consulting psychiatrists only. In this sample, 225 users (5.1 %) who made 41,334 consultations—that is, about 26.4 % of all consultations during the two-year period of the study—were classified as high service users. Considering that consultations and individuals can only be computed as discrete integers, the cut-off in the number of consultations had to be made at the closest value of the 95 percentile, explaining the 5.1 % instead of the exact 5.0 %. About 84.4 % of these high service users had at least one hospitalization and 76.9 % visited the emergency department during the study period. Another group of 3,007 users (68.2 %) made 108,523 consultations, and were classified as moderate users. About 36.5 % of the moderate users had at least one hospitalization and 21.5 % visited the emergency department. Finally, the remaining 1,175 users (26.7 %) made 6,516 consultations, and were classified as low users. About 12.7 % of the low users had at least one hospitalization and 3.8 % visited the emergency department. Table 1 summarizes number of consultations by group.

**Table 1:** Number of consultations with general practitioners and psychiatrists by individuals with substance use disorders (*n* = 4,407) between March 31, 2003 and April 1^st^, 2005

	Low service users	Moderate service users	High service users
	*n* = 1,175	*n* = 3,007	*n* = 225
Description of service users according to their number of consultations with general practitioners and psychiatrists	25^th^ percentile or lower	26–95^th^ percentile	95^th^ percentile or higher
Total number of consultations with general practitioners and psychiatrists	6,516	108,523	41,334
Range of consultations with general practitioners and psychiatrists	1–10	11–113	114–639
Proportion of consultations with general practitioners and psychiatrists	4.2 %	69.4 %	26.4 %
Average number of consultations with general practitioners and psychiatrists *(2-year period)*	5.5 ± 2.9	36.1 ± 23.3	183.7 ± 80.2
Average number of consultations with general practitioners and psychiatrists *(per month)*	0.2 ± 0.1	1.5 ± 1.0	7.7 ± 3.3
Average number of hospitalizations *(2-year period)*	1.3 ± 0.7	1.9 ± 1.4	4.5 ± 3.5
Average number of emergency department contacts *(2-year period)*	1.0 ± 0.3	1.6 ± 1.2	3.8 ± 2.8

Table 2 reveals that 63.1 % of high service users were men, and 52.9 % were 30 to 49 years old. The enabling factor of having consulted a medical professional other than GPs and psychiatrists was present for almost all (98.2 %) high service users. Concerning need factors, diagnosis of SUD (drug abuse, alcohol abuse and co-occurring alcohol and drug abuse) was given for 84.4 % of high service users. The prevalence of mental disorders was high among high service users: 94.2 % had an anxiety disorder, 64.9 % had a mood disorder, 52.0 % had a personality disorder, and 50.2 % had schizophrenia. In comparison with moderate service users and low services users, high service users differed significantly on four enabling factors and on all need factors: they were more likely to live in less populated areas and in a neighborhood with a higher percentage of 50–64 year olds, had more consultations with other medical professionals, used two or more ARC’s programs, and were more likely to report a diagnosis of schizophrenia, mood disorder, anxiety disorder, and personality disorder, to have a higher number of mental disorders and of comorbid physical conditions, and to present any pattern of comorbidity. In addition, as compared with low service users, high service users included a higher proportion of older people (predisposing factor) and lived in a neighborhood with a lower concentration of 50–64 years olds (enabling factor).

**Table 2:** Comparison between low or moderate service users and high service users of general practitioners and psychiatrists by individuals with substance use disorders (*n* = 4,407) between April 1, 2003 and March 31, 2005

Variable	Low service users^a^	Moderate service users^b^	High service users^c^	Low service users vs. High service users	Moderate service users vs. High service users
	*N* = 1,175	*N* = 3,007	*N* = 225	p^d^	p^e^
Predisposing factors					
Sex				0.065	0.119
Female	360 (30.6 %)	1 269 (42.2 %)	83 (36.9 %)		
Male	815 (69.4 %)	1 738 (57.8 %)	142 (63.1 %)		
Age group				0.002	0.424
up to 30 years	353 (30.0 %)	621 (20.7 %)	51 (22.7 %)		
30 to 49 years	613 (52.2 %)	1 702 (56.6 %)	119 (52.9 %)		
50-64 years	187 (15.9 %)	578 (19.2 %)	43 (19.1 %)		
65 years and above	22 (1.9 %)	106 (3.5 %)	12 (5.3 %)		
Enabling factors					
*Neighborhood characteristics*					
Population density (per km^2^)	7,218.6 ± 3,013.8	7,048.3 ± 2,877.3	6,494.9 ± 2,957.0	0.007	0.024
1^st^ quintile of neighborhood material deprivation	23.07 ± 21.56	21.02 ± 20.63	22.02 ± 20.11	0.582	0.567
5^th^ quintile of neighborhood material deprivation	23.54 ± 20.78	23.91 ± 20.30	23.54 ± 20.78	0.400	0.833
1^st^ quintile of neighborhood social deprivation	5.63 ± 9.09	5.12 ± 8.08	6.12 ± 8.96	0.547	0.147
5^th^ quintile of neighborhood social deprivation	50.60 ± 23.39	51.63 ± 23.35	50.40 ± 24.40	0.924	0.537
% of men	48.44 ± 1.69	48.40 ± 1.74	48.33 ± 1.65	0.462	0.650
% of persons aged up to 30	37.28 ± 2.76	37.16 ± 2.68	36.94 ± 2.65	0.163	0.347
% of persons aged 30-49	16.33 ± 2.69	16.21 ± 2.59	15.86 ± 2.42	0.048	0.117
% of persons aged 50-64	16.96 ± 1.61	17.06 ± 1.50	17.33 ± 1.50	0.010	0.033
% of persons 65 years and over	14.14 ± 3.33	14.26 ± 3.35	14.55 ± 3.20	0.158	0.305
% of recent immigrants	7.27 ± 3.41	7.02 ± 2.93	6.84 ± 2.88	0.149	0.472
Level of neighborhood social fragmentation	6.11 ± 4.43	6.07 ± 4.32	5.69 ± 4.28	0.282	0.295
Distance to the ARC (in km)	1.60 ± 0.87	1.65 ± 0.84	1.57 ± 0.93	0.624	0.212
Number of alcohol outlets with video lottery terminal	18.80 ± 8.13	18.80 ± 7.97	19.08 ± 8.21	0.699	0.675
Number of alcohol outlets without video lottery terminal	175.1 ± 83.41	171.4 ± 77.72	167.26 ± 83.02	0.295	0.528
*Health services utilization*					
Consulted other medical professionals	838 (71.3 %)	2 721 (90.5 %)	221 (98.2 %)	<.001	<.001
Number of programs used in the ARC				<.001	0.002
1	1 064 (90.6 %)	2 449 (81.4 %)	170 (75.6 %)		
2 to 3	110 (9.4 %)	531 (17.7 %)	48 (21.3 %)		
4 to 5	1 (0.0 %)	27 (0.9 %)	7 (3.1 %)		
Duration of care episode at the ARC				0.074	0.125
Less than one year	691 (60.9 %)	1 575 (55.7 %)	124 (58.5 %)		
One year	253 (22.3 %)	652 (23.0 %)	44 (20.8 %)		
Two years	93 (8.2 %)	305 (10.8 %)	19 (9.0 %)		
Three years	42 (3.7 %)	133 (4.7 %)	17 (8.0 %)		
Four years and more	55 (4.9 %)	165 (5.8 %)	8 (3.8 %)		
Duration in the program in the ARC (in years)				0.310	0.214
Less than one year	825 (70.2 %)	2 013 (66.9 %)	161 (71.6 %)		
One year	209 (17.8 %)	573 (19.1 %)	40 (17.8 %)		
Two years	70 (6.0 %)	199 (6.6 %)	14 (6.2 %)		
Three years	24 (2.0 %)	87 (2.9 %)	7 (3.1 %)		
Four years and more	47 (4.0 %)	135 (4.5 %)	3 (1.3 %)		
Need factors					
Diagnoses of substance abuse	221 (18.8 %)	1 528 (50.8 %)	190 (84.4 %)	<.001	<.001
Alcohol abuse	70 (6.0 %)	1 216 (40.4 %)	149 (66.2 %)	<.001	<.001
Drug abuse	156 (13.3 %)	868 (28.9 %)	171 (76.0 %)	<.001	<.001
Concomitant drug and alcohol abuse	17 (1.5 %)	493 (16.4 %)	122 (54.2 %)	<.001	<.001
Schizophrenia	8 (0.7 %)	334 (11.1 %)	113 (50.2 %)	<.001	<.001
Mood disorder	42 (3.6 %)	792 (26.3 %)	146 (64.9 %)	<.001	<.001
Anxiety disorder	199 (16.9 %)	1 965 (65.4 %)	212 (94.2 %)	<.001	<.001
Personality disorder	16 (1.4 %)	481 (16.0 %)	117 (52.0 %)	<.001	<.001
Number of mental disorders				<.001	<.001
0	954 (81.2 %)	842 (28.0 %)	4 (1.8 %)		
1	179 (15.2 %)	1 123 (37.4 %)	33 (14.7 %)		
2	40 (3.4 %)	737 (24.5 %)	60 (26.7 %)		
3	2 (0.2 %)	245 (8.2 %)	77 (34.2 %)		
4	0 (0.0 %)	60 (2.0 %)	51 (22.7 %)		
Number of physical comorbidity				<.001	<.001
0	895 (76.2 %)	1 433 (47.7 %)	56 (24.9 %)		
1 to 2	262 (22.3 %)	1 266 (42.1 %)	107 (47.6 %)		
3 to 5	18 (1.5 %)	283 (9.4 %)	49 (21.8 %)		
6 and over	0 (0.0 %)	25 (0.8 %)	13 (5.8 %)		
Concomitant substance and mental disorders	58 (4.9 %)	1 191 (39.6 %)	186 (82.7 %)	<.001	<.001
Concomitant substance and physical disorders	51 (4.3 %)	844 (28.1 %)	144 (64.0 %)	<.001	<.001
Concomitant mental and physical disorders	56 (4.8 %)	1 158 (38.5 %)	167 (74.2 %)	<.001	<.001
Concomitant substance, mental and physical disorders	11 (0.9 %)	660 (21.9 %)	142 (63.1 %)	<.001	<.001

^a^ 25^th^ percentile or lower for all consultations to GPs or psychiatrists

^b^ 26–94^th^ percentile for all consultations to GPs or psychiatrists

^c^ 95^th^ percentile for all consultations to GPs or psychiatrists

^d^ After Benjamini-Hocheberg’s correction, the significance level is fixed at *p* = .027

^e^ After Benjamini-Hocheberg’s correction, the significance level is fixed at *p* = .021

As presented in Table 3, needs factors were found to be the most significant predictors for high use of GP and psychiatrist services. Furthermore, only one enabling factor (receiving services at the ARC for three years prior to 2004) and four needs factors (having a diagnosis of schizophrenia, or mood, or anxiety or personality disorder) significantly increased the probability of being a high user of GPs and psychiatrists services.

**Table 3:** Generalized estimating equations on predictors of high service users of general practitioners and psychiatrists among individuals with substance use disorders (*n* = 4,407) between April 1, 2003 and March 31, 2005

	OR	SE	95 % CI		p^a^
			Lower	Upper	
Predisposing factors					
Sex (ref = female)	0.99	0.24	0.62	1.59	.975
Age group (ref = 29 years old and younger)					
30–49 years	1.05	0.33	0.55	1.99	.893
50–64 years	0.80	0.42	0.35	1.84	.601
65 years and older	4.14	0.69	1.06	16.08	.040
Enabling factors					
*Neighborhood characteristics*					
Population density (per square Km)	1.00	0.00	1.00	1.00	.130
1^st^ quintile of neighborhood material deprivation	0.99	0.03	0.92	1.05	.654
5^th^ quintile of neighborhood material deprivation	1.01	0.01	0.99	1.03	.436
1^st^ quintile of neighbourhood social deprivation	1.11	0.09	0.93	1.33	.255
5^th^ quintile of neighbourhood social deprivation	1.04	0.03	0.98	1.10	.160
% of men	0.79	0.26	0.47	1.32	.375
% of persons aged up to 30	2.09	0.46	0.85	5.16	.110
% of persons aged 30–49	3.05	0.44	1.28	7.29	.012
% of persons aged 50–64	3.89	0.56	1.31	11.56	.015
% of persons 65 years and over	1.60	0.38	0.75	3.40	.220
% of recent immigrants	1.03	0.21	0.68	1.54	.893
Level of neighbourhood social fragmentation	0.90	0.22	0.59	1.37	.613
Distance to the ARC (in km)	0.61	0.29	0.34	1.08	.090
Number of alcohol outlets with video lottery terminal	1.03	0.02	0.99	1.08	.101
Number of alcohol outlets without video lottery terminal	1.00	0.01	0.99	1.01	.984
*Health services utilization*					
Consulted other medical professionals	3.97	0.70	1.00	15.69	.050
Number of programs used (ref = one program)					
2 to 3 programs	0.64	0.27	0.38	1.07	.089
4 to 5 programs	0.37	0.75	0.08	1.60	.182
Duration of care episode at the ARC (ref = less than a year)					
One year	1.65	0.29	0.93	2.95	.088
Two years	1.00	0.37	0.49	2.06	.994
Three years	4.48	0.42	1.95	10.29	<.001
Four years or more	0.52	0.98	0.08	3.55	.504
Duration in the program in the ARC (ref = less than a year)					
One year	0.51	0.40	0.23	1.10	.087
Two years	0.89	0.44	0.37	2.12	.786
Three years	0.31	0.74	0.07	1.32	.114
Four years or more	0.63	1.05	0.08	4.90	.656
Need factors					
Diagnosis of substance abuse	0.95	0.94	0.15	6.03	.961
Alcohol abuse	2.41	0.72	0.59	9.85	.220
Drug abuse	3.41	0.64	0.98	11.87	.054
Concomitant drug and alcohol abuse	1.65	0.65	0.46	5.89	.443
Schizophrenia	4.74	0.21	3.14	7.16	<.001
Mood disorder	2.65	0.19	1.83	3.82	<.001
Anxiety disorder	2.34	0.30	1.29	4.25	.005
Personality disorder	3.42	0.26	2.07	5.65	<.001
Number of physical comorbidities (ref = no comorbidity)					
1 to 2	0.41	0.97	0.06	2.77	.363
3 to 5	0.67	1.00	0.09	4.78	.689
6 and over	9.86	0.95	1.53	63.70	.016
Concomitant substance abuse and mental disorders	0.48	0.55	0.16	1.39	.175
Concomitant substance abuse and physical disorders	1.40	0.72	0.34	5.76	.642
Concomitant mental and physical disorders	4.43	0.92	0.73	26.78	.105

^a^ After Benjamini-Hocheberg’s correction, the significance level is fixed at *p* = .007

## Discussion

This study used the Andersen model to examine a comprehensive set of predictors of high use of GP and psychiatrist services among individuals with SUDs using an ARC. The results of the study revealed that about 97 % of individuals attending the ARC had consulted a GP or psychiatrist in the two-year study period, for a mean of 1.5 consultations per month. Our results suggest that individuals with SUDs use not only ARC and emergency department services, but also require continuous care from GPs and psychiatrists. A small proportion of the sample (5 %), classified as high service users in accordance with the definition used in other studies, accounted for 26 % of total consultations to GPs or psychiatrists. One explanation is that, due to the complexity and chronicity of their needs, high service users tend to make more frequent use of services provided by emergency departments and hospital units than other service users. The use of these two services tends to inflate the overall number of consultations, where each contact with a medical professional in these settings counts as one consultation. The result that high users consulted a physician about twice a week on average may also be partially explained by other mental disorders, as well as chronic physical conditions, that require continuous medical monitoring, considering that about a half of our sample consulted both GPs and psychiatrists. Actually, before statistical correction, number of physical comorbidities was associated with being a high user. This clinically relevant result became statistically non-significant after correction. It is plausible that Benjamini-Hochberg’s procedure, although less conservative than Bonferroni’s correction, was too restrictive and increased false negative results (i.e. significant results being reported as non-significant). Another explanation of non-significance of physical comorbidities could be that mental disorders are so strongly associated with high service use that they mask the effects of physical illnesses in statistical analyses. Further research is needed to clarify if there is a real significant association between physical comorbidities and being a high user. The characteristics describing this group in the bivariate analyses need to be interpreted with caution, as a number of variables attributed to high service users are similar to the general profile of individuals with SUDs according to epidemiological studies, for example, being male, having co-occurring mental disorders, and living in densely-populated urban areas [1–6, 63, 64].

Multivariate analyses confirmed the hypothesis that higher needs associated with mental disorders determine higher use of GPs and psychiatrists by individuals with SUDs treated in an ARC. One enabling factor (attending the ARC for three years) suggests that high users of GPs and psychiatrists have more recurrent needs, which may require continuous and long-term care, of variable intensity, and follow-up in the community [65]. SUDs are considered chronic conditions for approximately half of individuals who receive treatment in ARCs [66]. This reality has led to the reorganization of health services in the US from crisis-oriented acute care to long-term recovery management approach for the SUD population, and to improvements in treatment effectiveness [67]. Currently, there are very few physicians affiliated with ARCs in Quebec and individuals with SUDs need to seek outside help from GPs and psychiatrists. This highlights the necessity to develop better coordination between medical services and ARCs in order to have a healthcare system that can adjust rapidly to the variable intensity and fluctuating needs of individuals with SUDs. Screening, Brief Intervention, and Referral to Treatment (SBIRT), addiction liaison-consultation teams in emergency settings, and cross-training programs for SUDs and mental disorders are examples of effective and efficient solutions for ensuring continuous care between primary medical care and ARCs [68, 69].

Concerning significant need factors, the present findings suggest that the likelihood of being classified as a high user of services increases with presence of mental disorders (schizophrenia, mood, anxiety, and personality disorders), as also documented in previous studies [70–72]. One study conducted in Montreal with patients who made multiple consultations to a hospital psychiatric emergency department also found that patients in the high service user group were significantly more likely to have schizophrenia [73]. Furthermore, an American study found that, among individuals with SUDs recruited from diverse settings (detoxification unit, primary care clinics, emergency departments, community), a high level of anxiety was associated with any treatment, and especially with the use of outpatient services delivered by psychiatrists and other medical doctors [26]. Another study among young Canadians with mental disorders, including SUDs, found that mood and personality disorders were associated with health services use [74]. The presence of borderline personality disorder, which often co-occurs with SUDs, is particularly known to be associated with high treatment use [75, 76]. Actually, the pattern of mental health services utilization found in this study is closer to the one found for all Quebec cases of personality disorders identified in the same databases by the public health agency chronic disease surveillance system [77]. As mental disorders also need to be addressed in the treatment context, ARC programs could benefit from improved collaboration between primary and specialist care services, and individual providers, in order to improve overall mental health care. Integrated treatment may also be efficient in helping individuals with co-occurring SUDs and mental disorders [78].

### Limitations

The present study was subject to some limitations. Although administrative databases are of value as a research tool, data were primarily collected for administrative purposes. They do not contain information on major determinants of health services use such as employment, personal income, and marital status. Furthermore, individuals who were excluded from the study due to lack of data on neighborhood variables (i.e. their LCSC territory) may have contributed particular characteristics to the study. For example, it is probable that they were homeless, which would explain why they did not have a LCSC territory attributed to them. Homeless individuals with SUDs represent a subgroup with specific characteristics; including them in this study may have impacted our findings. Some of their characteristics can be found in the control group of the Canadian and Montreal At Home/Chez Soi randomized pragmatic trial [79]. Another limitation is the low prevalence of SUDs reported in the RAMQ and Med-Echo databases, despite the fact that all participants were admitted to the ARC for SUDs. However, previous studies have indicated that only about 50 % of SUDs are detected during medical consultations or hospitalizations [80, 81]. Studies indicated that some GPs have negative attitudes toward individuals with SUDs and are reticent to treat these patients. Thus, in order to avoid taking on these patients, some physicians refrain from confirming a diagnosis of SUD during medical consultations [82–85]. An alternative explanation may be that services for SUDs in primary are scarce in Quebec, as in most other countries, so these patients have to seek help in specialized care [86, 87]. Some individuals attending the ARC could have been under the clinical threshold for a diagnosis of SUD, but nonetheless were experiencing important substance use problems [88]. Furthermore, a comparison between this research and previous studies is somewhat limited by methodological differences, for instance in terms of definitions of high service users, measured outcomes, and subpopulations. One final limitation concerns the gap between data collection and the availability of data for research. However, it is not unusual to observe a gap of approximately 10 years between data collection and publication of results, more so cases involving the use of administrative records [16]. Although our study data are not recent, there have been few changes in the Quebec healthcare system or in the epidemiologic profile of the Quebec SUD population to suggest that that the data collected and findings would not reflect the present reality of how services provided by GPs and psychiatrists are used by those individuals with SUDs who also receive services from the ARC.

## Conclusion

The originality of this study resides in the fact that the literature seldom investigates health services use outside of addiction-related programs, and is limited to clinical investigations of individuals with SUDs. Studies tend to focus instead on emergency departments or on service use within the ARC. This study is therefore one of the few that recognizes the fact that individuals with SUDs attending ARCs are also generally users of GPs and psychiatrists, and high users for a small proportion of them. This finding is in sharp contrast to the results of epidemiologic studies that have identified individuals with SUDs as low users of services in general. Furthermore, the findings are also original, as no previous study has compared high service users with other type of users among individuals with SUDs in terms of individual and neighborhood characteristics. Moreover, using administrative data gathered for the general population, and not for specific insured groups (e.g. Medicare for 65 years and older, and Medicaid for individuals with low income and limited resources), allows a better representation of the entire clientele treated in ARCs.

Overall, the findings of our study highlights that the needs of individuals with SUDs receiving services in ARCs are high, and that ARCs are dealing with a sub-population of individuals with SUDs whose addiction and mental health profiles are particularly complex. As the study confirms, these individuals are also high users of services provided by emergency departments and hospital units in the course of their consultations with physicians and psychiatrists. This situation underlines the necessity of providing a better fit between service organization and client needs. High users of services could benefit from more continuous and intensive care provided on a long-term basis, including comprehensive services with variable intensity and adjusted to fluctuating needs. ARC programs could also benefit from increased collaboration and integration with the mental healthcare sector.

## Abbreviations

ARC, addiction rehabilitation center; GEE, generalized estimating equations; GP, general practitioner; ICD-10, International Classification of Diseases – 10^th^ revision; ICD-9, International Classification of Diseases – 9^th^ revision; LCSC, local community services center; Med-Echo, *Maintenance et exploitation des données pour l'étude de la clientèle hospitalière;* RAMQ, *Régie de l’assurance-maladie du Québec*; SAS, Statistical Analysis System; SUD, substance use disorder
